# The vibrating reed frequency meter: digital investigation of an early cochlear model

**DOI:** 10.7717/peerj.1333

**Published:** 2015-10-13

**Authors:** Andrew Bell, Hero P. Wit

**Affiliations:** 1 John Curtin School of Medical Research, Australian National University, Canberra, Australia; 2 Department of Otolaryngology/Head and Neck Surgery, University of Groningen, Groningen, The Netherlands

**Keywords:** Coupled oscillators, Frahm reed, Cochlea, Traveling wave, Resonance

## Abstract

The vibrating reed frequency meter, originally employed by Békésy and later by Wilson as a cochlear model, uses a set of tuned reeds to represent the cochlea’s graded bank of resonant elements and an elastic band threaded between them to provide nearest-neighbour coupling. Here the system, constructed of 21 reeds progressively tuned from 45 to 55 Hz, is simulated numerically as an elastically coupled bank of passive harmonic oscillators driven simultaneously by an external sinusoidal force. To uncover more detail, simulations were extended to 201 oscillators covering the range 1–2 kHz. Calculations mirror the results reported by Wilson and show expected characteristics such as traveling waves, phase plateaus, and a response with a broad peak at a forcing frequency just above the natural frequency. The system also displays additional fine-grain features that resemble those which have only recently been recognised in the cochlea. Thus, detailed analysis brings to light a secondary peak beyond the main peak, a set of closely spaced low-amplitude ripples, rapid rotation of phase as the driving frequency is swept, frequency plateaus, clustering, and waxing and waning of impulse responses. Further investigation shows that each reed’s vibrations are strongly localised, with small energy flow along the chain. The distinctive set of equally spaced ripples is an inherent feature which is found to be largely independent of boundary conditions. Although the vibrating reed model is functionally different to the standard transmission line, its cochlea-like properties make it an intriguing local oscillator model whose relevance to cochlear mechanics needs further investigation.

## Introduction

The vibrating reed frequency meter, also called the Frahm frequency meter, is an analogue instrument with a long history of use as a cochlear model, extending back to at least Békésy (1960) and Wilson (1992). The model is of particular interest because when its bank of tuned reeds are elastically coupled, most simply with a rubber band, and when the system is energized with an oscillating magnetic field, traveling waves can be seen running from the high-frequency reed at one end to the low-frequency reed at the other—in just the same way as waves in the mammalian cochlea are observed to run along the basilar membrane from base to apex in response to a sound stimulus.

The vibrating reed frequency meter has not attracted further attention since it was last investigated by Wilson, and has long been superseded by models of the cochlea based on the electrical transmission line (Allen, 1977; Allen, 1980; Allen & Sondhi, 1979; De Boer, 1996; Nobili, Mammano & Ashmore, 1998; Peterson & Bogert, 1950; Shera, 2003; Zweig, 1976; Zwislocki, 1950; Zwislocki, 2002). With refinements to allow for nonlinearity, short-wave behaviour, added dimensions, and active mechanics, transmission line models have come to form the basis of cochlear mechanics (see also Duifhuis, 2012; Epp, Verhey & Mauermann, 2010; Ni et al., 2014; Verhulst, Dau & Shera, 2012; Shera, 2015; Zweig, 2015).

Why then revisit an obsolete piece of measuring equipment? One motive was set out in an earlier work (Bell, 2012), where it was shown that a bank of tuned resonators could produce a traveling wave remarkably similar to that seen in the cochlea. Incentive also comes from recognising that it is easy to simulate the vibrating reed system using modern computers. The Frahm frequency meter can be modelled numerically as an array of passive harmonic oscillators, graded in frequency and elastically coupled, and driven in parallel by a sinusoidal force. Computational tools allow the various effects of forcing, coupling, and frequency gradient to be examined in detail. It is also of interest to see if previous work done with mechanical analogues can be replicated, to explore the system more closely, and to compare results with recent findings in cochlear mechanics.

### The vibrating reed frequency meter

The vibrating reed frequency meter, also called the Frahm frequency meter after its inventor, Hermann Frahm (Daffron & Greenslade, 2013), comprises a graded set of tuned metal reeds, typically 11 or 21, which are arranged to vibrate in response to an oscillating magnetic field or mechanical vibration. They are rugged analogue instruments useful for measuring the frequency of a mains supply or an engine’s rotational speed, and are still available today even though digital devices have largely supplanted them.

**Figure 1:**
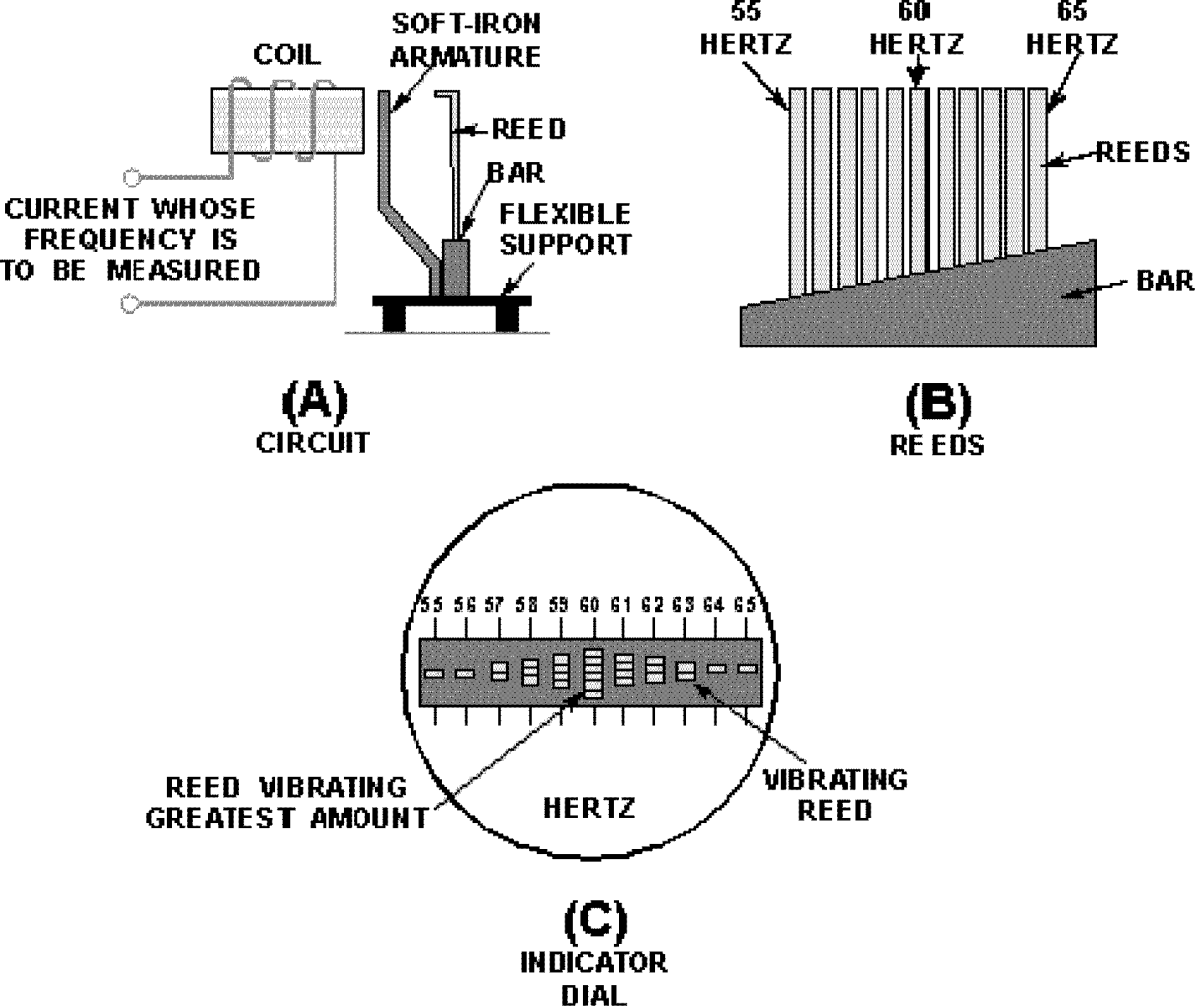
Basic features of the vibrating reed frequency meter. (A) Arrangement by which the reeds are excited by an oscillating magnetic field. (B) Graded tuning of the reeds, in this case 11 reeds ranging from 55 to 65 Hz. (C) End-on view of the reeds, showing maximum vibration of the middle reed, indicating a mains frequency of 60 Hz. Image credit: from Fig. 1-47 of Hicks (1998), *Introduction to circuit protection, control, and measurement*, NAVEDTRA 14175, US Navy.

Frahm frequency meters are essentially a side-by-side array of tuning forks which operate on the principle of mechanical resonance (Bakshi & Bakshi, 2009; Hicks, 1998). A simplified diagram is shown in Fig. 1, where 11 reeds ranging from 55 Hz to 65 Hz are shown (Fig. 1B). When the reeds are mechanically excited at a certain frequency, say 60 Hz, the reed with matching natural frequency will tend to vibrate the most. In practical terms, if the instrument is to measure the frequency of the mains supply, a coil is used to generate an oscillating magnetic field which in turn attracts a soft iron armature, producing an oscillating mechanical force on all the reeds (Fig. 1A). Incidentally, because the alternating current creates magnetic attraction twice each cycle, the reeds are actually tuned to double the indicated frequency. Devices designed to measure engine speed are held directly against the engine, in which case no scaling is necessary. The instrument’s dial is an end-on view of the reeds, and the reed vibrating with the highest amplitude indicates the frequency of the supply (Fig. 1C). Simplicity and robustness are the instrument’s major attributes.

Historically, the vibrating reed frequency meter is noteworthy for its early use as a cochlear model by Békésy (1960) and Wilson (1992). These workers used the instrument’s graded bank of tuned reeds to represent the cochlea’s progressively tuned array of resonant elements, and added an elastic band threaded between the reeds to provide coupling.

The major feature of this early modelling work was the appearance of an eye-catching traveling wave, which always moved from the high frequency reed to the low frequency one. A traveling wave appeared as soon as the instrument was energised by an oscillating magnetic field, whether the reeds were coupled or not. However, a persistently running traveling wave was produced only when the reeds were coupled; in this case the waves continually ran from “base” to “apex” as long as the field was switched on. This distinctive behaviour was taken by Békésy to be a good facsimile of what he saw in the cochlea in response to sound, and he spent time manipulating the system to better understand how it worked and refine his traveling wave theory of cochlear excitation.

This paper begins by establishing a single analytic equation to describe the vibrating reed system and then solves it numerically. An initial step was to confirm the analogue modelling done by Wilson (1992), which involved 21 reeds tuned at 0.5 Hz intervals from 45 Hz to 55 Hz and coupled using a rubber band. The numerical calculations are directly compared with Wilson’s analogue model, matching the parameters he used as closely as possible. Finally, to investigate some unexpected features, the system is extended to 201 oscillators and higher frequencies (1–2 kHz), its impulse responses are examined, and its energy fluxes analysed. The conclusion reached is that the vibrating reed system is an interesting example of a local oscillator model which, despite its simplicity, can replicate a surprisingly large number of cochlear properties, including the appearance of features only recently recognised, such as a secondary peak beyond the main peak (Zweig, 2015), amplitude ripples, and frequency clustering (Shera, 2015).

### Békésy

**Figure 2:**
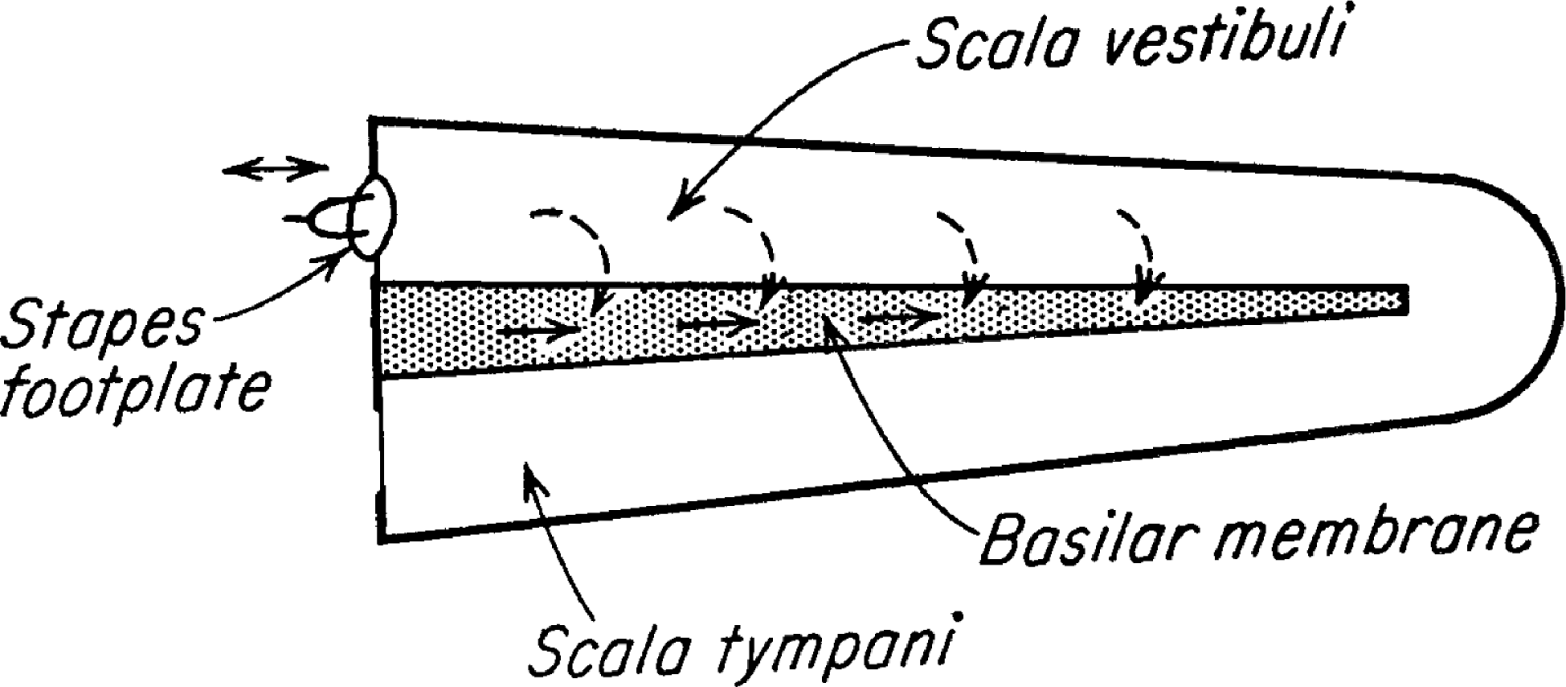
Békésy’s diagram of the two ways by which sound can stimulate the cochlea. The *dashed arrows* are multiple pathways though the fluid, meaning that the stimulus is acting in parallel and reflecting what happens in a resonance model. The *solid arrows* represent a serial stimulus along the basilar membrane, and indicate what he assumed took place in a traveling wave model. The original question he asked was which of the two energy paths dominates, and the vibrating reed system offers, in simple form, a way of examining the question. Image credit: adapted from Fig. 3 of Békésy, 1956, Simplified model to demonstrate the energy flow and formation of traveling waves similar to those found in the cochlea, *Proceedings of the National Academy of Sciences of the United States of America* 42: 930–44.

The Frahm frequency meter plays an important role in Békésy’s classic book (p. 514 ff. of Békésy, 1960). In the Frahm device he found a good model for the traveling wave behaviour he had seen in human temporal bones. He had observed that traveling waves always travel from base to apex, irrespective of where sound entered the cochlea. He called the propagation of waves towards an apical vibration source “paradoxical” and sought a model that could explain such nonintuitive behaviour (he went so far as to describe the behaviour as “unfortunate” (p. 510 of Békésy, 1960), possibly because traveling wave theory tends to create the impression that sound energy is deposited sequentially as it propagates along the basilar membrane, see Fig. 2). Noting that the problem was a hydromechanical one, and that “hydromechanics [is] a field in which plausible reasoning has commonly led to incorrect results” (*ibid*., p. 510), he first looked at a transmission line model, but found it lacked the features he was looking for. It is apparent, he said, that the cochlea is not simply a high-pass transmission line, for “the driving forces are exerted not just on the first section alone but on every individual section” (p. 515)—in other words he took the sound stimulus to act in parallel, not series—so he felt compelled to look elsewhere for a suitable model to explain paradoxical waves.

At this point he introduces the Frahm frequency meter, a device he had long been acquainted with (from as early as 1928; p. 413, pp. 494–495) and which was, he thought, superior to the transmission line in having parallel inputs to the reeds. More importantly, he found that this model could support paradoxical wave propagation under various conditions of stimulation.

Békésy’s vibrating reed system was a set of about 100 metal strips, each carrying a small mass on top to adjust tuning. As it happened, Békésy’s main concern was the form of the coupling—stiff or compliant, viscous or nonviscous—and not so much the effect of tuning gradients. So for many experiments the reeds were tuned equally, and he adjusted coupling by varying the position of an interwoven strand of rubber or by immersing the reeds in oil or water (his Fig. 12-42). Nevertheless, he did set up a system in which the reeds had a uniform gradient in resonant frequency (his Fig. 12-47), and he reports that traveling waves with an amplitude and phase distribution “similar to those in the human cochlea were obtained” (p. 521). Under appropriate conditions, paradoxical waves were seen. Then, with a returning focus on serial excitation, Békésy claims (p. 522) that a section near the stapes must receive energy and transmit it to the more yielding parts, as in his Fig. 12-43A. This statement seems to reflect, implicitly if not explicitly, a way of thinking in which energy propagates sequentially along the partition, an idea that continues to recur in traveling wave models.

Békésy is now almost finished with his treatment of the tuned reed system. There is another use of tuned reeds later on in his book (pp. 548–549) where he again considers cochlear frequency analysis and concludes from slow-motion filming of tuned reeds that there is in fact “a large overlap” between resonance and traveling wave theories. However, despite the promising cochlea-like properties of the Frahm device, he moved on to “living skin” models involving a neural law of contrast, a way by which he thought sharper tuning could be produced.

In terms of evaluating energy flows in the cochlea, the vibrating reed system offers a way of addressing the question, and this is another reason for revisiting it here. In Fig. 2 there are two sets of arrows—one depicting a parallel stimulus path and the other a serial path. In the first case, sound reaches all the sensing cells almost simultaneously via the fluid (dashed arrows), and with the vibrating reed this corresponds to parallel forcing of the reeds by the imposed external field. But if the stimulus travels along the membrane, the energy will move serially along the chain (solid arrows), as in the elastic band coupling the vibrating reeds. Békésy explains that under the parallel model, fixation of one section of basilar membrane will have no effect on neighbouring sections; however, for the serial stimulus, fixation is expected to prevent further propagation (p. 525 of Békésy, 1960). Later, he professed agnosticism between which path represents the actual state of affairs in the living cochlea (Wever, Lawrence & Békésy, 1954). There is still discussion today about the various stimulus paths in the cochlea (Recio-Spinoso & Rhode, 2015; Shera, Tubis & Talmadge, 2004; Van der Heijden, 2014; Van der Heijden & Versteegh, 2015), so it is of interest to see what happens in a system such as the vibrating reed in which the parallel path predominates.

The analysis here suggests there is still much to be learnt from the vibrating reed system. This paper shows that as well as reproducing a traveling wave, the vibrating reed system can display surprisingly intricate behaviour, such as clustering and other response irregularities, which are relevant to current investigations (Shera, 2015; Temchin & Ruggero, 2014; Zweig, 2015).

### Wilson

Following Békésy, the next appearance of the vibrating reed frequency meter in a cochlear context was the work of Wilson (Wilson, 1983; Wilson, 1992). His first investigation was reported in a conference abstract in which he describes how, when a rubber thread was intertwined between the reeds of a Frahm reed meter and the reeds stroboscopically illuminated, a traveling wave could be seen to move from the high frequency end to the low frequency end.

These observations largely confirm what Békésy saw, but they go further in describing distinctive phase plateaus in which the phase of all the low-frequency reeds accumulates a lag of *π*, 3*π*, or 5*π* radians. Wilson’s conclusion was that, if a stiffness-controlled phase advance of *π*/2 at low frequencies was factored in, the vibrating reed model gives an outcome “consistent with published basilar membrane data.” He later published a fuller description of the system (Wilson, 1992) in which quantitative findings of both amplitude and phase were presented, not just the qualitative descriptions which Békésy provided. It was provided within a general discussion of cochlear mechanics involving Helmholtz, Békésy, Gold, and others and set out to demonstrate that the Frahm system can give a fairly detailed account of key cochlear observations.

Wilson’s findings can be almost completely confirmed computationally, as the results below illustrate. As will be shown, the advantage of the digital model is that the parameters can be instantly adjusted and fine details of the system investigated.

## Methods

An equation describing the vibrating reed system is constructed and solved numerically. The results of the calculations are directly compared with the analogue modelling of Wilson (1992) using matching parameters wherever possible.

Wilson used a Frahm frequency meter with 21 reeds tuned from 55 Hz at one end (reed 1) to 45 Hz at the other (reed 21). Here, the coupled oscillator model used in previous work (Wit & Van Dijk, 2012; Wit, Van Dijk & Manley, 2012) was adapted to replicate this situation. In brief, the present model is a simplified, real (not complex), passive version of the earlier work of Vilfan & Duke (2008), who assembled a chain of viscoelastically coupled active oscillators to simulate the effect of an external tone on the hearing organ of the lizard.

Other workers have used similar coupled chains of tuned elements to simulate the cochlear mechanics of lizards (Gelfand et al., 2010; Wit, Van Dijk & Manley, 2012) and mammals (Duke & Jülicher, 2003; Epp, Verhey & Mauermann, 2010; Van Hengel, Duifhuis & Van den Raadt, 1996; Wit & Van Dijk, 2012). However, it is worth emphasising that all these approaches (except for that of Authier & Manley, 1995) employed active oscillators, based on Van der Pol oscillators, which carry extra but unknown dynamical effects. Here, the complication of active dynamics is absent, and only the intrinsic passive behaviour of the oscillators is examined.

A chain of 21 elastically coupled passive oscillators can be considered as an array of mutually coupled damped harmonic oscillators with displacement *x_j_*(*t*), so that

(1) \begin{eqnarray*} {x}_{j}^{{\prime\prime}}+\gamma {\omega }_{j}{x}_{j}^{{\prime}}+{\omega }_{j}^{2}{x}_{j}={f}_{0}\sin \omega t+\kappa ({x}_{j-1}-2{x}_{j}+{x}_{j+1}),\hspace{1em}\text{for }j=1,2,\ldots 21, \end{eqnarray*}

where *γ* is the damping, *κ* is the spring constant between neighbouring oscillators, *ω* the angular frequency, and *t* time. The resonant frequencies of the reeds were set by having *ω_j_*/2*π* = 55, 54.5, …45 (rather than separately specifying mass and stiffness). The forcing strength *f*_0_ is arbitrary, since the system is linear, and was here set to 1,000 (any value of *f*_0_ gives the same result, apart from a scale factor). The last bracketed term in Eq. (1) is the coupling force acting between adjacent oscillators and is proportional to the difference in instantaneous displacement of the oscillators, just as a spring or rubber band would provide. The coupling is varied by letting *κ* assume a range of real values. (As a point of comparison, Vilfan & Duke (2008) considered the lizard ear as an array of coupled oscillators in which the coupling was made to be either dissipative or reactive, or both, by letting it take on various complex values; they also allowed the oscillators to be passive or active by specifying the damping to be positive or negative.) For the first oscillator, the term in brackets was set to *x*_2_–*x*_1_, and for the last oscillator to *x*_20_–*x*_21_. Starting conditions were ${x}_{j}(0)={x}_{j}^{{\prime}}(0)=0$ for all *j*. The parameter *γ* was initially set to 0.014, to provide a quality factor, *Q* = 1/*γ*, for the uncoupled oscillators comparable to Wilson’s reeds (which had *Q* values—half-power bandwidth relative to resonant frequency—of about 70). The size of the elastic coupling used by Wilson is unknown, but values of *κ* from 0 to 5,000 appear to cover the range he used and values were chosen to best mimic his results. A rubber band provides both coupling and damping, so combinations of *κ* and *γ* that best reproduced Wilson’s results were obtained by trial and error. These combinations were approximated by *γ* = 3.37 ⋅ 10^−6^*κ* + 0.00553.

The set of 21 coupled differential equations was solved with the NDSolve procedure (with “automatic” option) in Mathematica v.10 (Wolfram Research, Long Hanborough, UK) for a 5-second time interval.

In a second phase of the investigation, the number of oscillators was extended to 201 or more, with the same governing equations. The natural frequencies of the oscillators accorded with Greenwood’s frequency–position map for the human ear (Greenwood, 1990) over a distance of 16.5–18.5 mm from the apex in 0.01 mm steps (the width of a hair cell). This produced an almost linear range of natural frequencies from 1.5 to 2 kHz in about 2.5 Hz steps. The coupling constant, *κ*, here ranged from 0 to 400 (smaller than before because the relative frequency difference was now smaller). The *Q* values remained roughly the same, this time about 40. Again, the Mathematica procedure NDSolve was implemented to calculate the response over a 100 ms interval (more than 100 cycles) due to forcing at 1.75 kHz, or over 50 ms when the impulse responses of the oscillators were being calculated. The steady state amplitude reached during the last 3 cycles was the key measure plotted.

### Results with 21 reeds

#### Formation of traveling waves and phase plateaus

**Figure 3:**
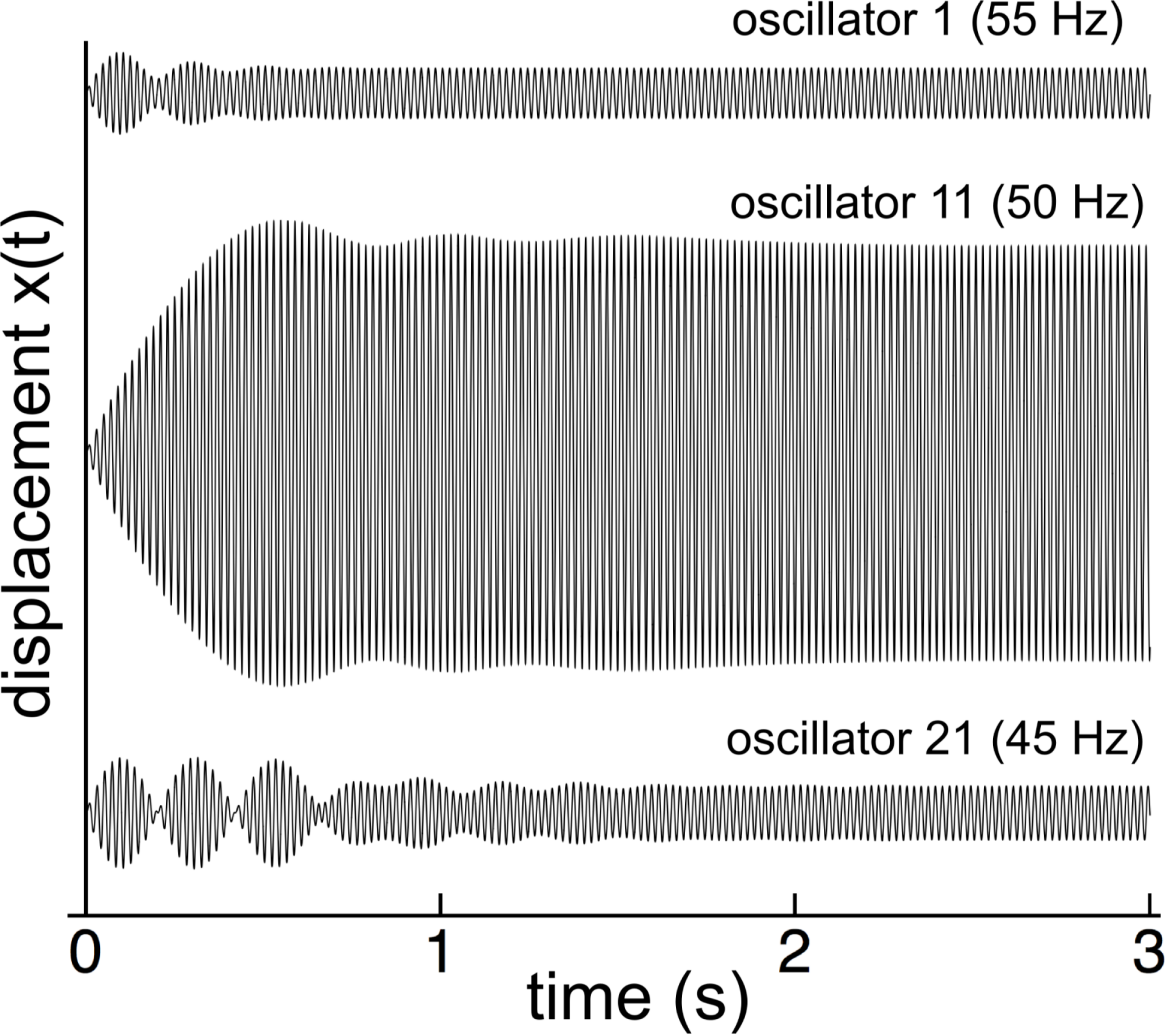
Switch-on transients for 3 reeds (55, 50, and 45 Hz) for 50 Hz excitation. After 2 seconds, all the reeds have settled into steady state oscillation at 50 Hz in step with the external driving force.

The first part of Wilson’s investigation was to impose a 50 Hz oscillating magnetic field on all the reeds and measure their amplitude after the initial switch-on transient had faded away. Similarly, the numerical modeling (with *γ* = 0.014 and *κ* = 2,500) showed that, when forced at 50 Hz, the reed system went through a complex initial transient period, settling down after a number of seconds (Fig. 3).

**Figure 4:**
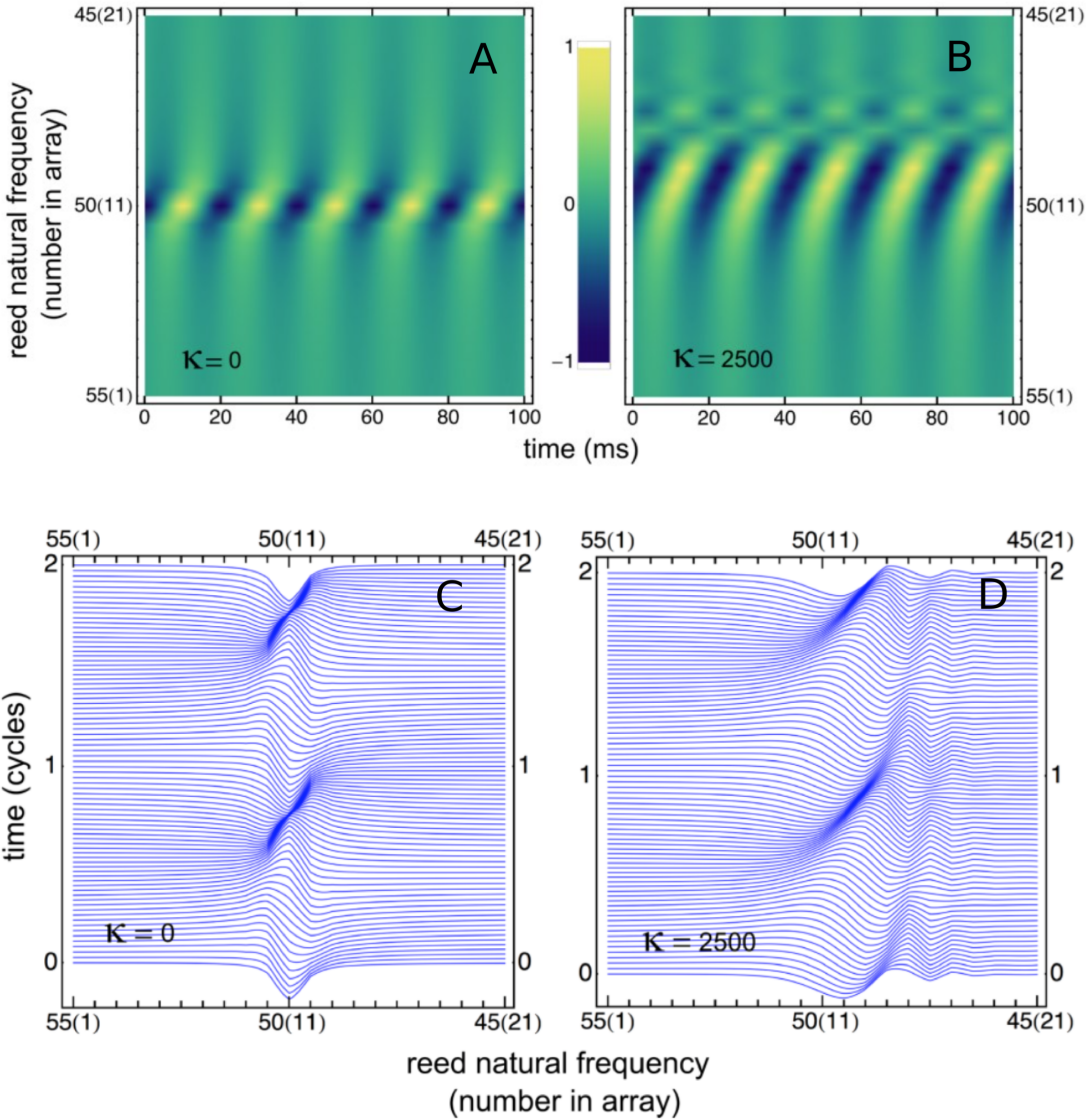
Variation of reed displacement with time. (A, B) Displacement over 5 cycles, plotted as density, for 21 reeds driven at 50 Hz for uncoupled reeds (A) and coupled reeds (B). (C, D) Waterfall plot of the instantaneous displacement of the reeds over 2 cycles. Traveling waves appear to form, most clearly for the coupled case.

When steady state was achieved, all the reeds oscillated at the driving frequency of 50 Hz, but with different amplitudes and phases, as shown in Fig. 4A and 4B for both uncoupled and coupled reeds (*γ* = 0.014 in both situations). As can be seen, coupling creates less sharp tuning (broader peaks), a shift in peak amplitude towards reeds with lower natural frequencies, and, especially for the lower frequency reeds, a larger phase delay with respect to the driving force.

**Figure 5:**
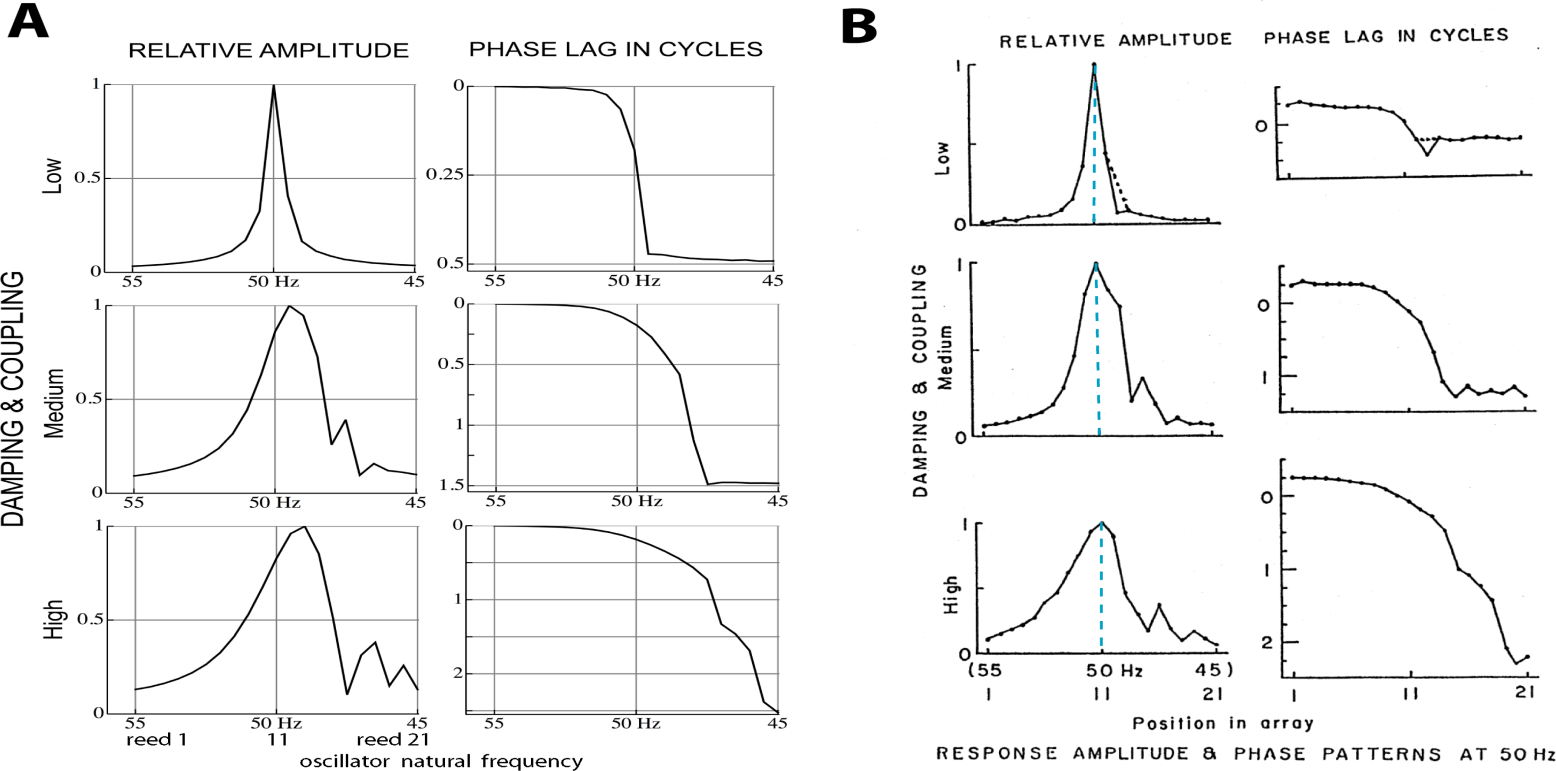
Normalised amplitude and phase of 21 reeds driven by a 50 Hz external force. (A) Calculated numerically for three different combinations of coupling strength *κ* and damping ratio *γ* (150, 0.006; 2,200, 0.013; and 4,600, 0.021). The peak shifts to lower natural frequencies as coupling increases (note the reversed frequency axis). Secondary peaks appear on the low frequency side. (B) Amplitudes and phases as measured by Wilson (1992). Vertical dashed lines mark the 50 Hz driving frequency, which coincides with the peak at all levels of coupling. The discrepancy with the calculated result is discussed in the text. Image credit: from Wilson (1992), Cochlear mechanics, *Advances in the Biosciences* 83:71–84, with permission of the author.

**Figure 6:**
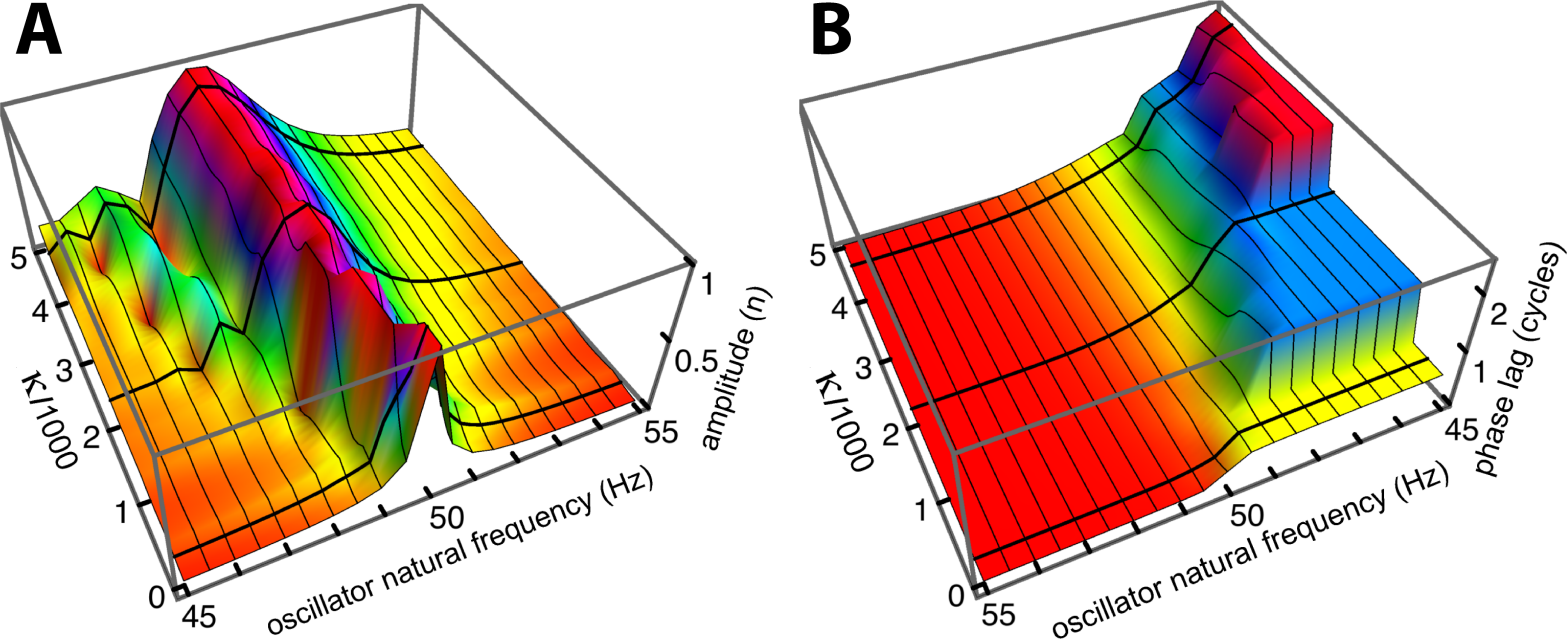
Normalised amplitude (A) and phase (re the 55 Hz oscillator, B) of the 21 oscillators. The calculations are for 51 combinations of *κ* and *γ*, where *γ* = 3.37.10^−6^*κ* + 0.00553. The coupling parameter *κ* ranges from 0 to 5,000 in steps of 100, and the damping ratio *γ* is adjusted accordingly. The three thick black lines in each panel follow values of *κ* of 150, 2,200, and 4,600, and give the amplitude and phase profiles shown in Fig. 5A.

A similar result can be obtained by off-setting plots of instantaneous displacement vs time, giving the waterfall plots of Figs. 4C and 4D. Horizontal lines in the figure show reed displacements at 0.5 ms instants, and it is clear that a wave of displacement appears to travel through the array in the direction of the lower frequency reeds. The “traveling wave” is not so visible in the uncoupled situation, but clearly evident in the coupled case. Relative amplitudes of the 21 reeds as a function of position in the array are plotted in Fig. 5A. Here, combinations of *κ* and *γ* were chosen that best reproduced Wilson’s results (Fig. 5B). Also shown are the phase lags of the reeds, plotted relative to the reed with the highest natural frequency (55 Hz). Expanding the range of coupling parameters to 51 combinations of *κ* and *γ*, the resulting amplitudes and phases are shown in Fig. 6.

Comparing Figs. 5A and 5B, it is seen that the simulations give similar results to Wilson’s measurements, including the growth, as coupling increases, of a secondary peak a little distance to the right (lower natural frequency) of the main peak. Phase delays also increase as the coupling is made stronger, reaching 2.5 cycles for the lowest frequency oscillator when (*κ*, *γ*) = (4,600, 0.021). The phase behaviour was confirmed by calculating the argument of the Fourier transform of *x_j_*(*t*) for the steady state situation. After unwrapping the phase to eliminate 2*π* phase jumps, the same phase curves were produced (results not shown). One difference is apparent between Wilson’s experimental findings and the calculated responses: Wilson found that the amplitude peak occurred for a driving frequency identical to the natural frequency of the reed, whereas the simulations find that the peak occurs at a natural frequency slightly below the driving frequency. More light on this discrepancy comes from a closer examination of Wilson’s data, which is done in the next section.

#### Frequency response of a single reed

**Figure 7:**
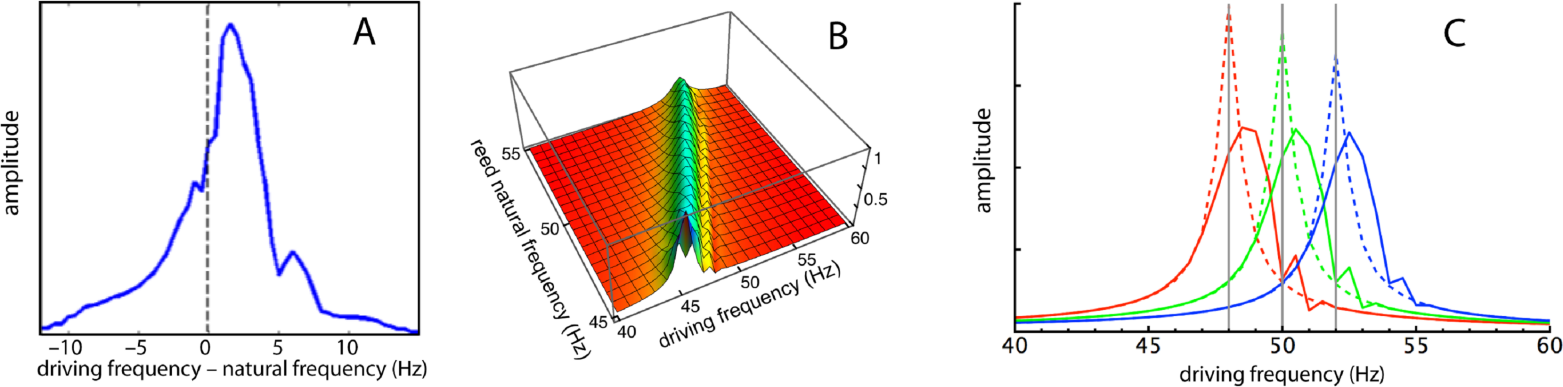
Frequency responses of specific reeds. (A) Average frequency response of four reeds as measured by Wilson (1992). Here the responses of the reeds (47, 48, 49, and 50 Hz) have been aligned in terms of their natural frequencies, so that 0 Hz corresponds to each reed’s natural frequency and the amplitudes are 4-point averages as the driving frequency was moved from 10 Hz below to 15 Hz above the natural frequency. (B) Calculated response of all 21 reeds for (*κ*, *γ*) = (2,200, 0.013) as driving frequency was increased from 40 to 60 Hz. (C) Three slices through the middle plot showing frequency responses of reeds with natural frequencies of 48, 50, and 52 Hz. Dotted lines show responses for *κ* = 0 (uncoupled).

To this point the analysis has focused on the response of the reeds in the spatial dimension, that is the amplitude profile along the set of reeds when all are excited at a common driving frequency, the situation reported by Wilson (1992) in his Fig. 1A. This approach is useful in depicting the relative amplitudes, the occurrence of phase lags, and of course the formation of a “traveling wave”, an apparent movement over time of the peak response from the high frequency reeds to the lower frequency ones. In the cochlea, however, the usual situation is the observation of a single point on the basilar membrane having a fixed characteristic frequency, while the rest of the membrane and its “off-frequency” elements are hidden from view. This second situation is captured by plotting the frequency response curve, which is the response of an observed point to a wide range of stimulating frequencies. Wilson studied this condition by selecting a particular reed and measuring its displacement amplitude as the global driving frequency was varied from 38 to 62 Hz. The raw results are presented in his Fig. 2B for both amplitude and phase. However, Wilson’s data points are noisy and a pattern is hard to discern; moreover the legend unfortunately carries a mistake in labelling (reeds 11, 13, 15, and 17 must have corresponding natural frequencies of 50, 49, 48, and 47 Hz to have the sequence of −3 dB points shown in the phase plots). The error is corrected in Fig. 7A, and here, to reduce noise and improve clarity, the amplitudes are averages over the four sets of measurements.

For comparison, the simulation results are presented in Figs. 7B and 7C, and it can be seen that the profiles are similar. In all cases, the peak response appears at a forcing frequency above the natural frequency of each reed. This also applies to Wilson’s data (Fig. 7A here), suggesting that the earlier plots made by him (Fig. 5B), where the peak occurred at a natural frequency identical to the forcing frequency, probably involved a degree of experimental error.

### Results with 201 reeds

**Figure 8:**
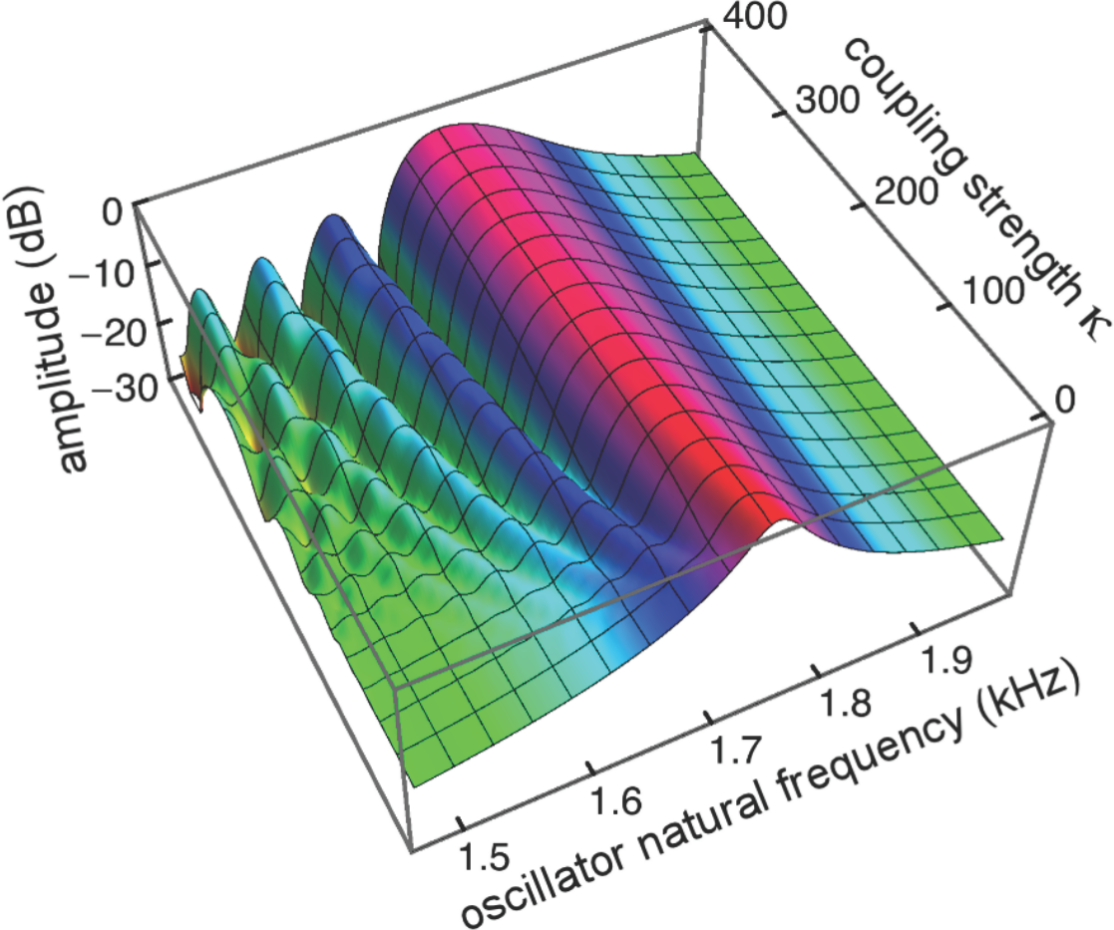
Amplitude of 201 elastically coupled oscillators in response to global forcing at 1.75 kHz. The natural frequency of the oscillators ranges from 1.5 to 2 kHz; the coupling strength parameter, *κ*, varies from 0 to 400 in steps of 10.

To investigate the behaviour of the vibrating reed system in more detail, the number of reeds was increased to 201, and the range of their natural frequencies was shifted to 1.5 kHz–2 kHz. The result of calculating the amplitude of each reed after 100 ms of forcing is shown in the plot of Fig. 8, which shows the envelope of the oscillators’ displacement on a logarithmic scale as coupling is varied from 0 to 400.

Coupling shifts the peak to reeds of lower natural frequency and, in this spatial plot, introduces a distinctive succession of ripples on the side of the peak with lower natural frequencies. As coupling increases, the spacing between the major peak and the ripples widens.

**Figure 9:**
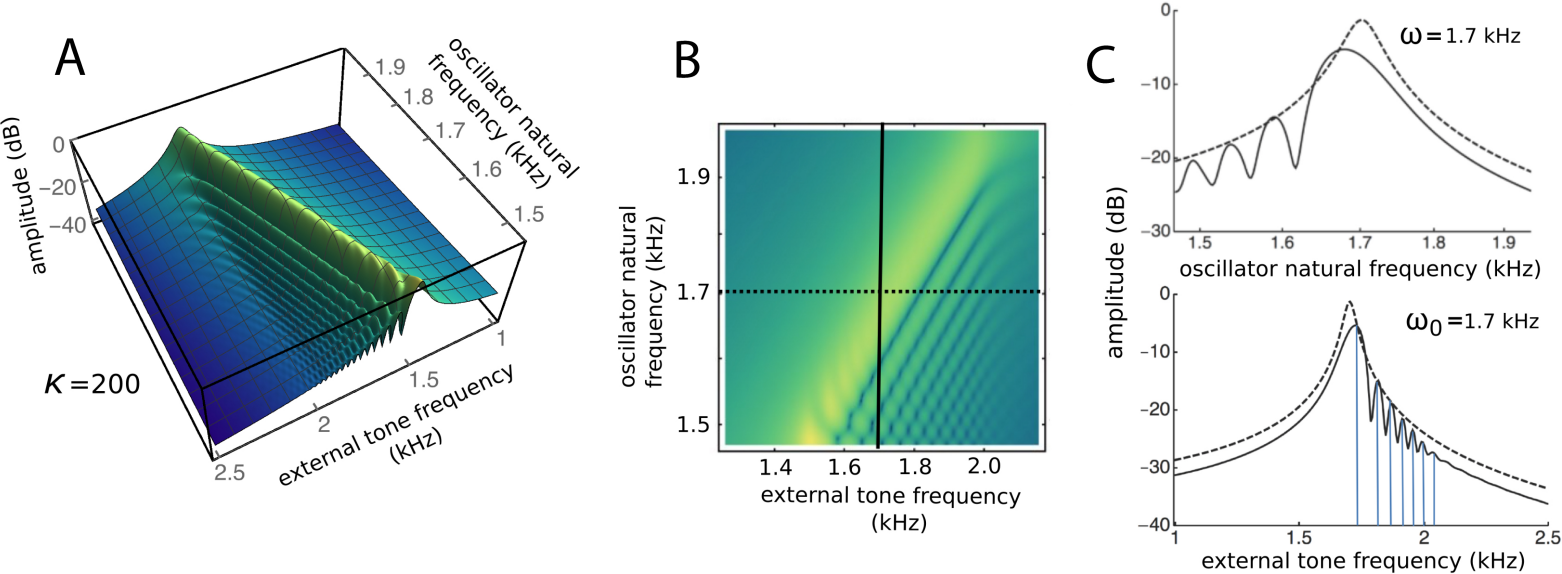
Frequency response of the 201-reed system. (A) Amplitude of a set of 201 oscillators of natural frequency 1.5 to 2 kHz in response to driving frequencies of 1 to 2.5 kHz. (B) Plan view of A showing the regularity of the ripples. (C) Sections through B at the continuous line (upper) and dotted line (lower). In both, dashed lines represent the uncoupled case. The coloured vertical lines mark the position of peaks, which, for the chosen value of kappa, occur at a ratio of 1.06 (first two lines) and 1.03 (subsequent lines).

To draw out the source of the ripples, the responses of the same 201 oscillators were calculated as the global driving frequency was varied from 1 to 2.5 kHz. The result for *κ* = 200 is shown in Fig. 9A. The figure required 8 h calculation time on a MacBook Pro. It is helpful here to recognise that driving frequency and natural frequency track in opposite directions: ripples on the side of low natural frequency (for say a constant 1.7 kHz driving frequency) correspond to ripples on the high-frequency side of the forcing frequency (for an oscillator of 1.7 kHz natural frequency). More simply, the opposing trends can be seen in Fig. 9C as a property of the two orthogonal transects made in the plan view of Fig. 9B. The vertical lines in Fig. 9C mark the positions of the peaks, and it is of interest that, for the coupling parameter chosen, the second peak occurs at a frequency ratio of about 1.06 to the first, after which the other peaks appear at regular intervals of about 1.03.

### Factors underlying the ripples

**Figure 10:**
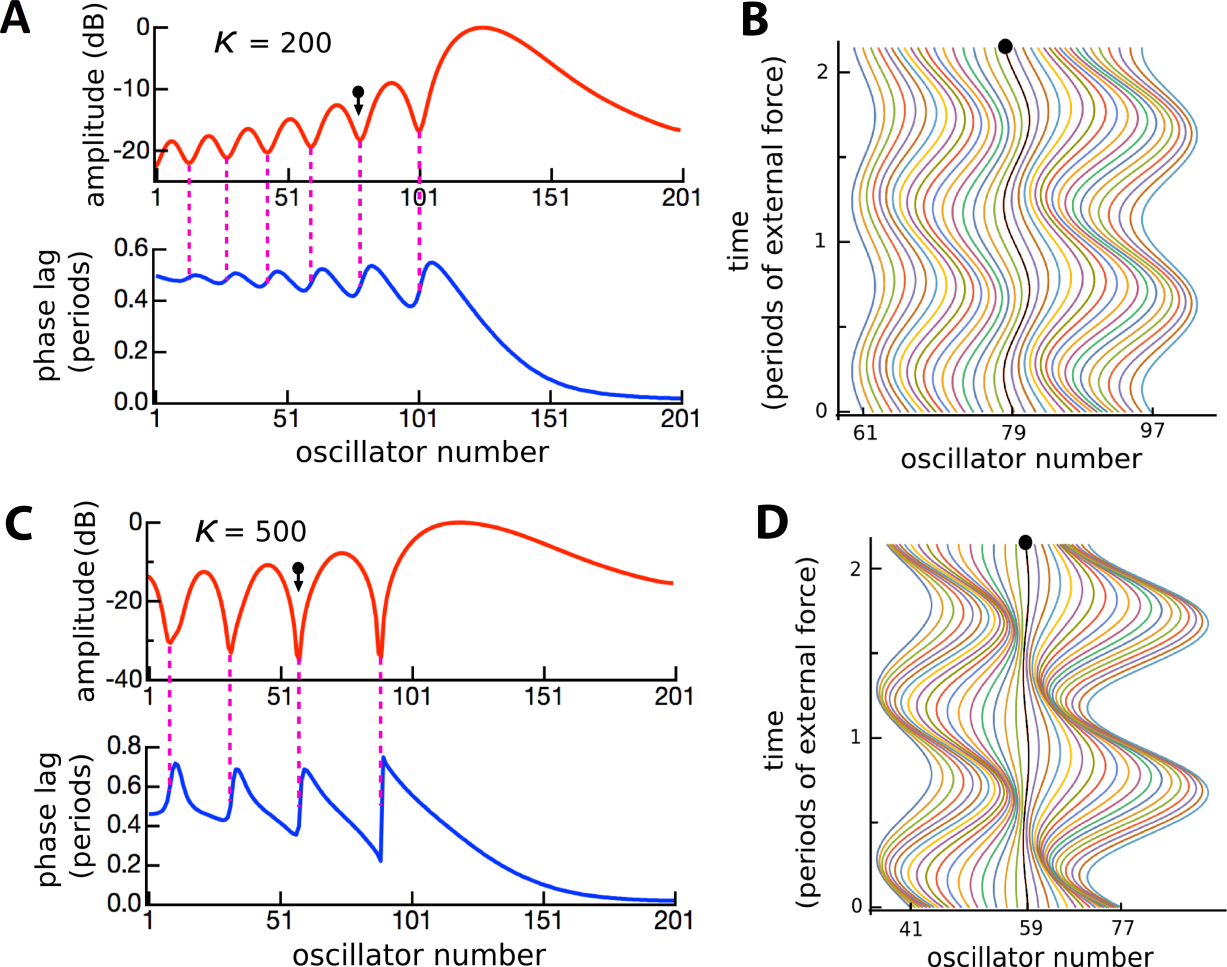
Ripples defined by phase transitions. Amplitude and phase (A) of the displacement (B) of 201 coupled oscillators of natural frequency 1.5 to 2 kHz in response to a driving frequency of 1.8 kHz, plotted over 2 cycles (*κ* = 200). (C, D) The same except for *κ* = 500. The ball and arrow mark an amplitude minimum. Phase is calculated with respect to the external force. For clarity, the waterfall plots are shown for a restricted range.

The factors responsible for the formation of the ripples were of interest and prompted further investigation. The amplitudes and phases of the oscillators were calculated, and the results are plotted in Fig. 10. It is clear that the amplitude minima are accompanied by rapid phase transitions. The number of ripples decreased and their size increased as the coupling becomes stronger, reinforcing the results of Fig. 8. It was also found that the number of ripples and their size depended on the relative frequency spacing of adjacent oscillators (becoming smaller as more oscillators filled a given frequency range), and that ripple amplitude depended on damping: as *γ* increased, the ripple pattern became shallower (results not shown).

**Figure 11:**
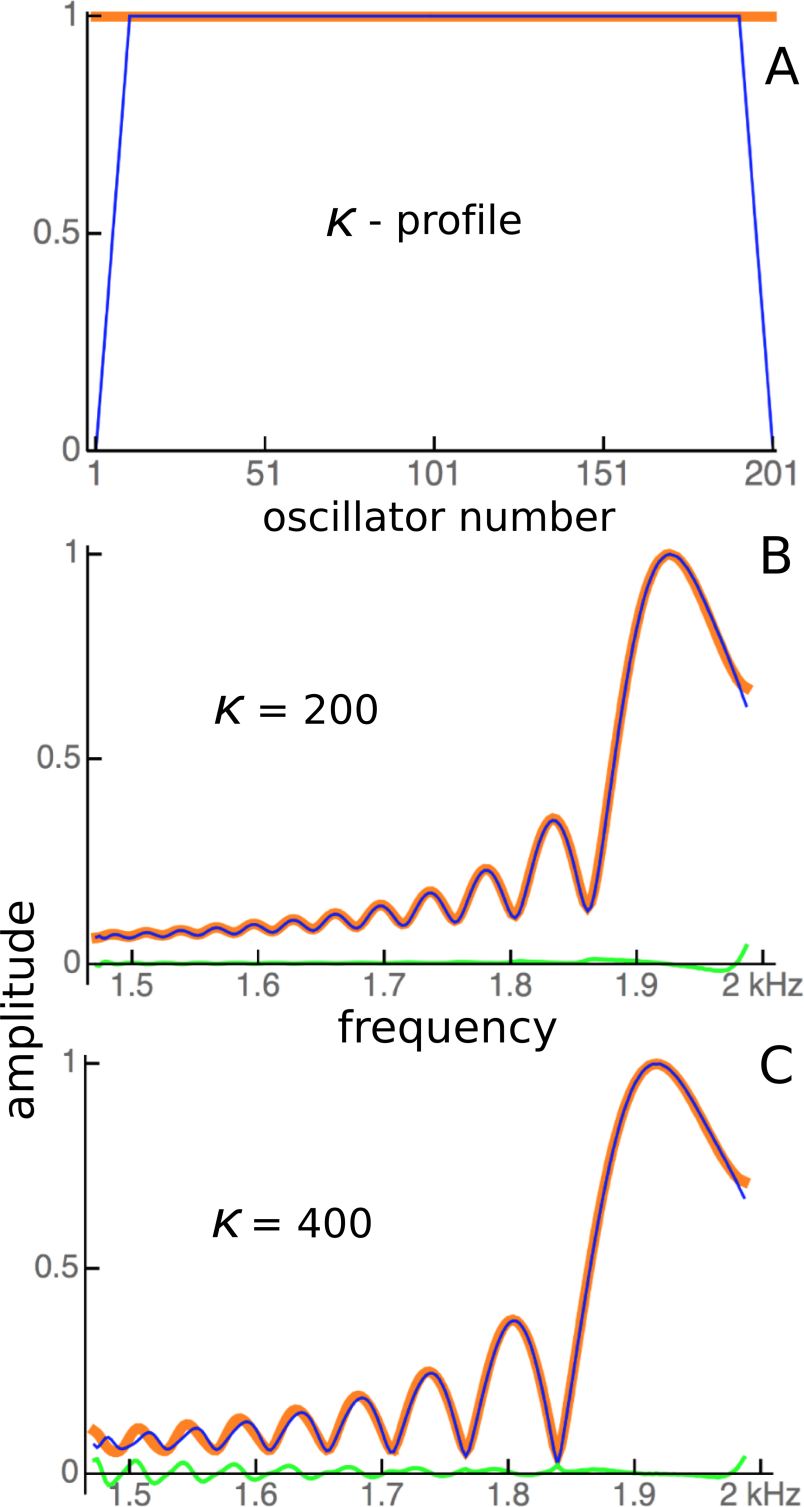
The effect of changing boundary conditions on the response of 201 coupled reeds. (A) The profile of the coupling parameter, *κ*, was changed from being constant along the entire array, including at the ends (red), to an arrangement where coupling was zero at the ends and increased progressively over 10 reeds to a fixed maximum (blue). (B) For a maximum *κ* of 200, the responses under the two conditions are shown with the red and blue lines, with the difference between them in green. (C) As for B, but for *κ* = 400.

In a number of cochlear models, a regular finding is that the boundary conditions of the basilar membrane—the impedance at the oval window and helicotrema—can give rise to oscillations in the frequency response further along the partition (Epp, Verhey & Mauermann, 2010; Shera, 2015; Van Hengel, Duifhuis & Van den Raadt, 1996). The standard explanation is that the ripples are due to reflection of traveling waves so that, when encountering an impedance discontinuity, a traveling wave will be reflected and give rise to interference between forward- and backward-propagating components. The outcome is a standing wave with characteristic ripples and a system of nodes and antinodes. To test whether the ripples in the vibrating reed system were due to wave interference, the effect of a drastic change in boundary conditions was examined. The coupling parameter *κ* was either left constant along the entire length of the array, or *κ* for the outermost reeds was set to zero and then progressively increased over a short sequence of 10 reeds, as shown in Fig. 11A. The difference in frequency response between the two conditions was plotted for two values of *κ* (Figs. 11B and 11C).

Figure 11 demonstrates that changes in coupling gave rise to small perturbations in responses at the edges, but over the majority of the system there were only minor differences between one profile and another. Altering the *Q* (by adjusting *γ*) also had no effect on the position of the ripples. However, the ripples did become more prominent when coupling was increased or damping was decreased. Figure 11 provides evidence that the ripples are largely the result of a local interaction between a single reed and the forcing field surrounding it, and that there is little energy propagating from one reed to another. Impedance-matching at the ends does not appear to be a major factor at work in the vibrating reed system. The predominance of local-oscillator behaviour is examined further in the following section.

#### Energy sources and sinks

When the vibrating reed system is uncoupled, the driving energy from the oscillating magnetic field causes each reed to vibrate, and this vibrational energy is then dissipated in resistive damping (the size of which controls the *Q* of the reed). When the reeds are coupled, however, some vibrational energy will propagate to neighbouring reeds via the elastic band, so that a “traveling wave” will carry some energy from high frequency reeds to low frequency ones. This situation relates to the original question raised by Békésy of whether hair cells were excited in parallel by energy transmitted through the cochlear fluids, or by energy passing serially along the basilar membrane (see Fig. 2). In the coupled vibrating reed system, both paths are present. The results of changing the coupling profile (Fig. 11) seem to indicate that, for the range of parameters selected, the amount of energy carried along the system was small compared to local excitation.

**Figure 12:**
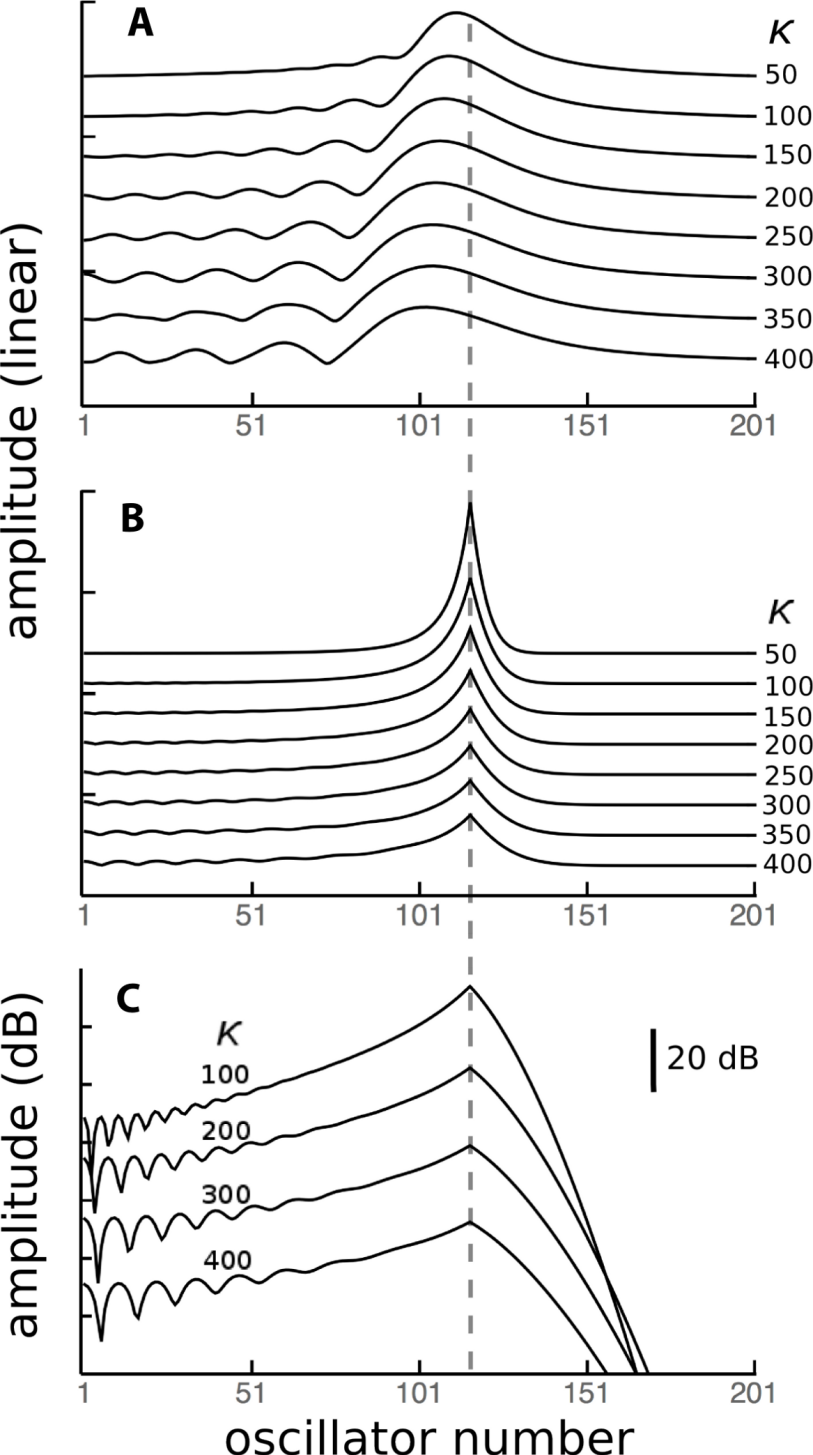
Contribution of a local energy source to global responses. Because the vibrating reed system is linear, the global response of all 201 reeds when driven in parallel at 1.75 kHz (A) can be considered to be a superposition of the responses of each of the reeds driven individually. One of these responses, for reed 116 of natural frequency 1.75 kHz (dashed line) is shown in (B), and it shows that the response of this reed is largely due to its external forcing, with very little activity passing to neighbouring reeds. When the response is plotted logarithmically (C), it is clear that energy passing towards lower frequency reeds (leftwards, in the direction of the traveling wave) is less attenuated than energy moving to higher frequency reeds (to the right, as a reverse traveling wave).

In an effort to confirm this, use was made of the linearity of the Frahm system, recognising that its total response is essentially the superposition of the responses of each of the individual reeds. By driving only one reed (not, as usual, all of them), the relative amount of energy dissipated locally, measured in terms of that reed’s amplitude of vibration, could be compared to the energy transmitted to neighbouring reeds (measured as their vibration amplitudes). The result of driving a single central reed is shown in Fig. 12.

As a point of reference, Fig. 12A shows, for various coupling strengths, the standard vibrating reed system with all reeds driven with an oscillating force of 1.75 kHz. The familiar response envelopes are shown on a linear scale. The following Figs. 12B and 12C show, on linear and logarithmic scales respectively, the response obtained by driving only a single reed, the one with a natural frequency of 1.75 kHz. The plots indicate how far vibration propagates along the chain of reeds.

It can be seen that the extent of energy propagation is limited, with a 20 dB loss occurring within about a dozen reeds in the high frequency direction, and perhaps a few times that number in the low frequency direction. The slopes on either side of the peaks in Fig. 12C correspond to attenuation rates of about 160 dB and 900 dB/oct respectively. That is, most of the energy is locally absorbed and transmission distance is relatively small, particularly in the high frequency direction. Interpreted another way, the energy inherent in any traveling wave would be relatively small compared to the energy delivered by external forcing, and that carried by a reverse traveling wave would be even smaller.

### Further properties of the ripples

**Figure 13:**
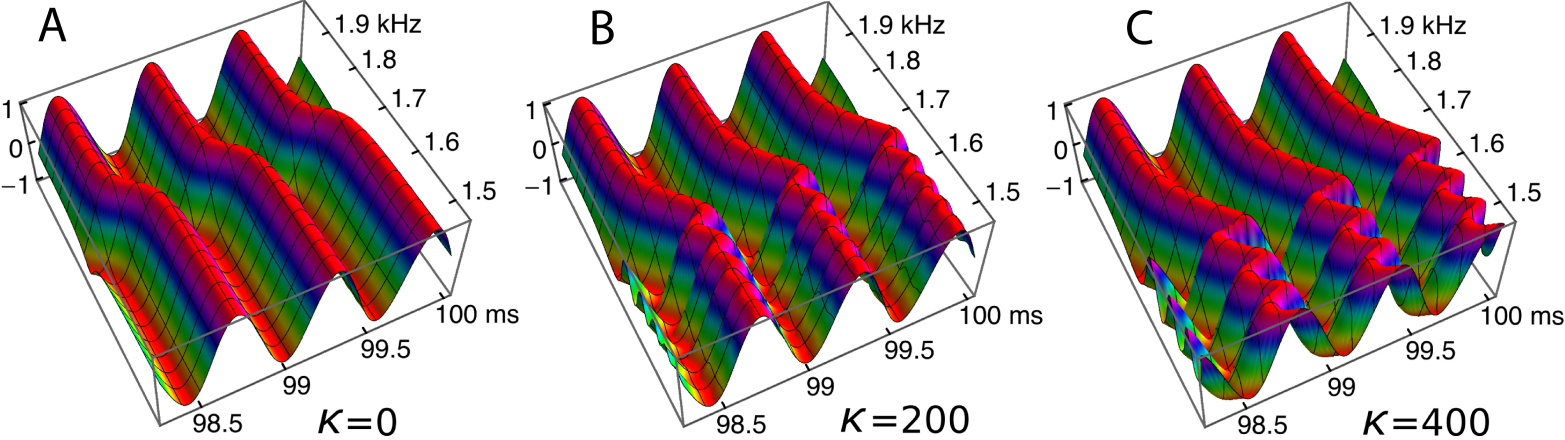
Bands of phase variation in an array of 201 oscillators in response to a driving frequency of 1.75 kHz. The plots show, for three coupling constants, *κ* = 0 (A), 200 (B), and 400 (C), the normalised amplitude during the last three cycles of a 100 ms simulation. Each oscillator has settled down to a regular cycle, but there are systematic bands of phase along the array—in the spatial dimension—with a periodicity of about 60 Hz (B) or 80 Hz (C).

Ripples have been shown to be an arresting phenomenon arising from interaction between the forcing field and the reeds. This section returns to the ripples and explores them from a somewhat different perspective. Figure 10 showed regular variations in phase and amplitude, and here this picture is confirmed and extended. The ripples are this time plotted (Fig. 13) as a 3-D plot of the amplitude profile of 201 oscillators after steady state had been reached, and, unlike Fig. 10, the maximum displacements have been normalised to show the phase variations more clearly. A refinement shown in the figure is the way in which the phase variations increase as the coupling parameter increases (Figs. 13A–13C). In the case of no coupling, as depicted in Fig. 13A, every oscillator is effectively in step with its neighbours after 100 ms of simulation. However, when the oscillators are elastically coupled—Figs. 13B and 13C—there are now bands of phase variation along the array with a period of about 60–80 Hz.

**Figure 14:**
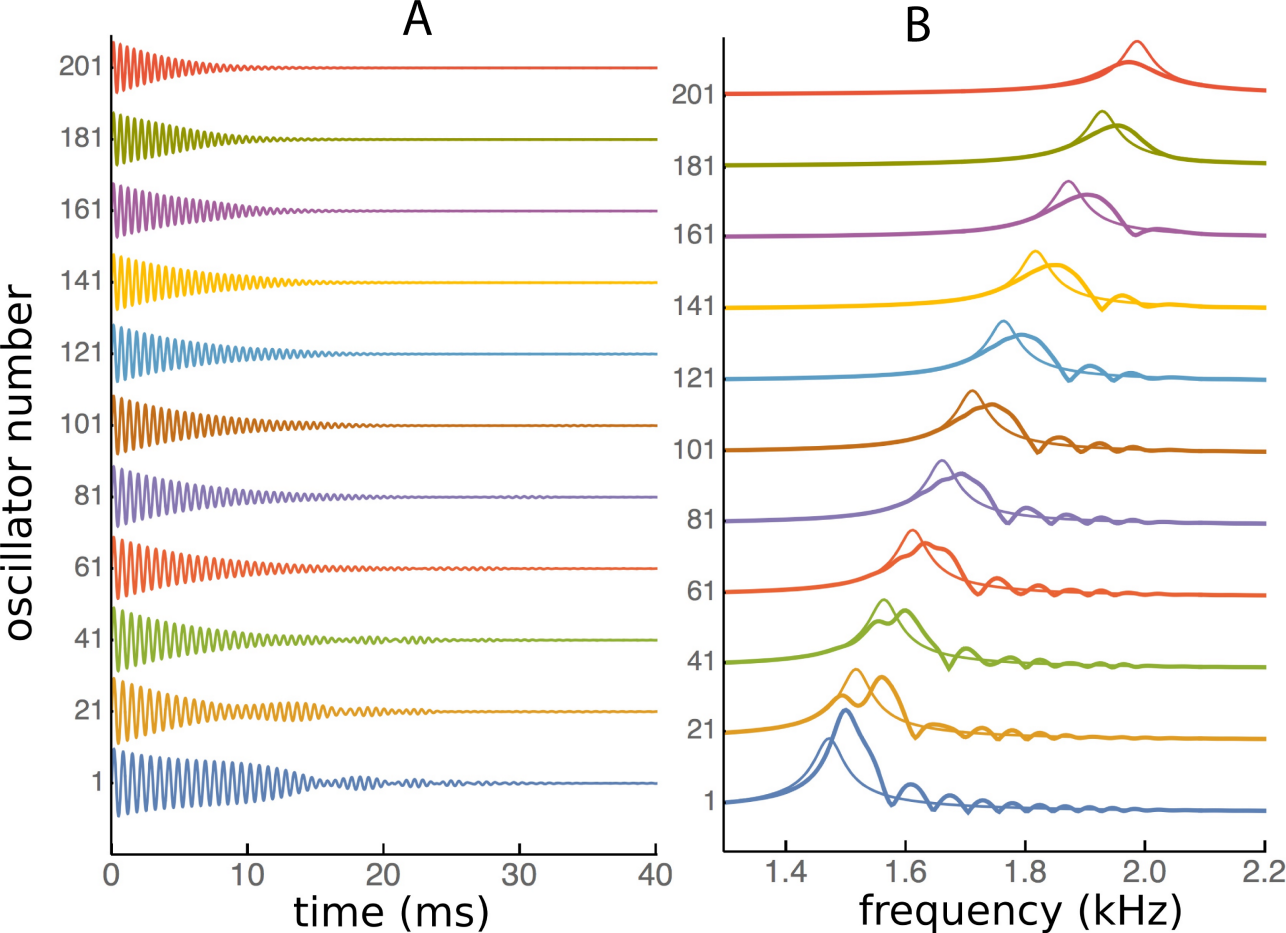
Impulse responses in the time and frequency domains. (A) Impulse response of 11 individual oscillators in a set of 201 elastically coupled oscillators ranging in frequency from 1.5 kHz (oscillator number 1) to 2 kHz (number 201). (B) The corresponding frequency response derived by Fourier transformation. The thin line shows the uncoupled response. Once again it is seen that the peak response occurs at a higher frequency than the natural frequency, and there is a series of frequency peaks, whose spacing, as in Figs 8–11, occurs at ratios of about 1.05 for *κ* = 200.

The ripples in the spatial domain should also be evident in the frequency domain, so impulse responses of each reed were calculated and Fourier transformed. The impulse responses were calculated from Eq. (1) by setting *f*_0_ = 0 and the starting conditions *x_j_*(0) = 0 and ${x}_{j}^{{\prime}}(0)=1$ for all *j*. This was done both for the standard Frahm device with natural frequencies of 45 to 55 Hz (21 reeds) and for the higher frequency case with 201 oscillators ranging from 1.5 to 2 kHz. The impulse responses for the higher frequency set are displayed in Fig. 14, together with the corresponding frequency domain plots.

The time domain waveforms in Fig. 14 show, at least for the lower frequency reeds, a distinct waxing and waning of amplitude. The corresponding frequency-domain plots show that the waxing and waning derives from the presence of multiple frequencies—the set of regular ripples—which beat together. Once again, the frequency ratios between the peaks have values of about 1.06 for the first two peaks and about half that for subsequent ripples (when a *κ* of 200 was used).

**Figure 15:**
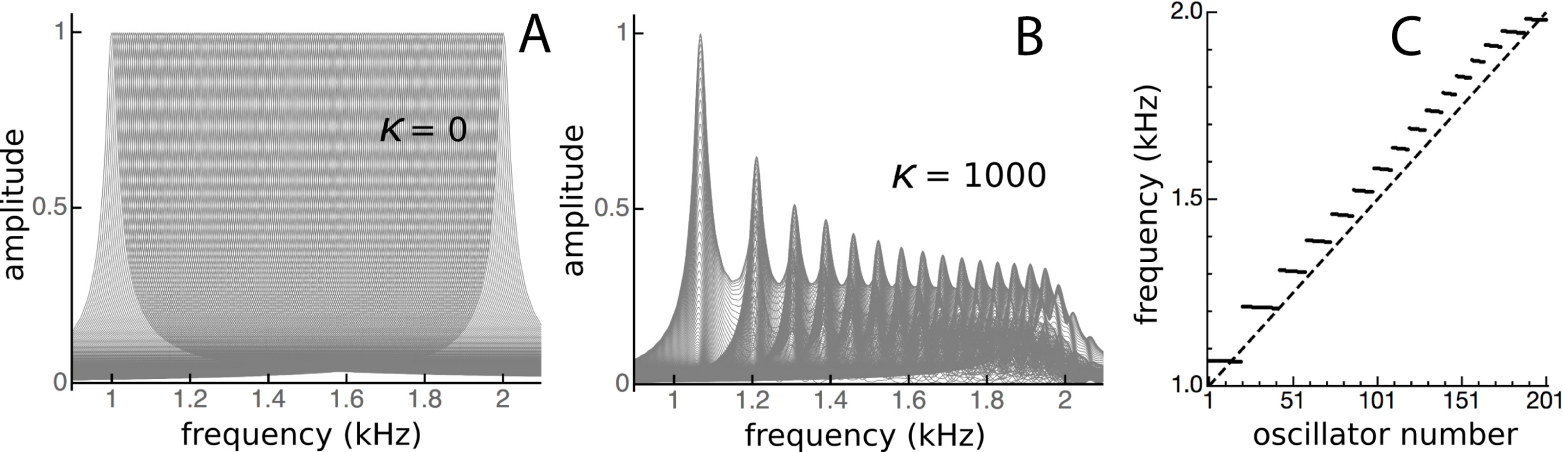
Clustering of oscillators as elastic coupling between them is increased. Overlapping spectra of the impulse responses of an array of 201 passive damped harmonic oscillators for equally spaced frequencies from 1 to 2 kHz and *κ* of zero (A) and 1,000 (B). When the frequencies of the highest peaks in the responses in B are plotted (C), distinct frequency plateaus can be seen for *κ* = 1,000. The dashed line is for *κ* = 0.

Note here that Fig. 14 shows the impulse responses of individual reeds. To calculate the response of the whole system to an impulse applied simultaneously to every reed, all the individual responses would need to be added together, which again makes use of the system’s linearity. Figure 15 shows the result of superimposing the spectra of the impulse responses from each of the oscillators. Superposition is not the same as addition, but visually it provides a good indication. Uncoupled, the spectra are uniform (Fig. 15A), but under coupled conditions the spectra cluster into distinct frequency plateaus (Figs. 15B and 15C). The stronger the coupling, the larger the separation of the plateaus, which, for *κ* = 1,000, gave a ratio of about 1.1 at the lower frequencies and became increasingly finer at higher frequencies. Similar to the waxing and waning exhibited by a single reed (Fig. 14), the plateaus indicate that the entire system is expected to show waxing and waning when subjected to a global impulse.

## Discussion

Numerical modelling has shown that the vibrating reed frequency meter can be replicated as a set of passive harmonic oscillators elastically coupled and driven by an alternating force. The simulations in large measure confirm the work of Wilson (1992), done decades ago with his analogue device. The system performs frequency analysis, and produces a range of interesting features such as traveling waves, asymmetric tuning curves with a peak on the side of higher driving frequency (or of lower natural frequency), phase plateaus, secondary peaks, closely spaced frequency ripples, clustering, and an impulse response that waxes and wanes.

**Figure 16:**
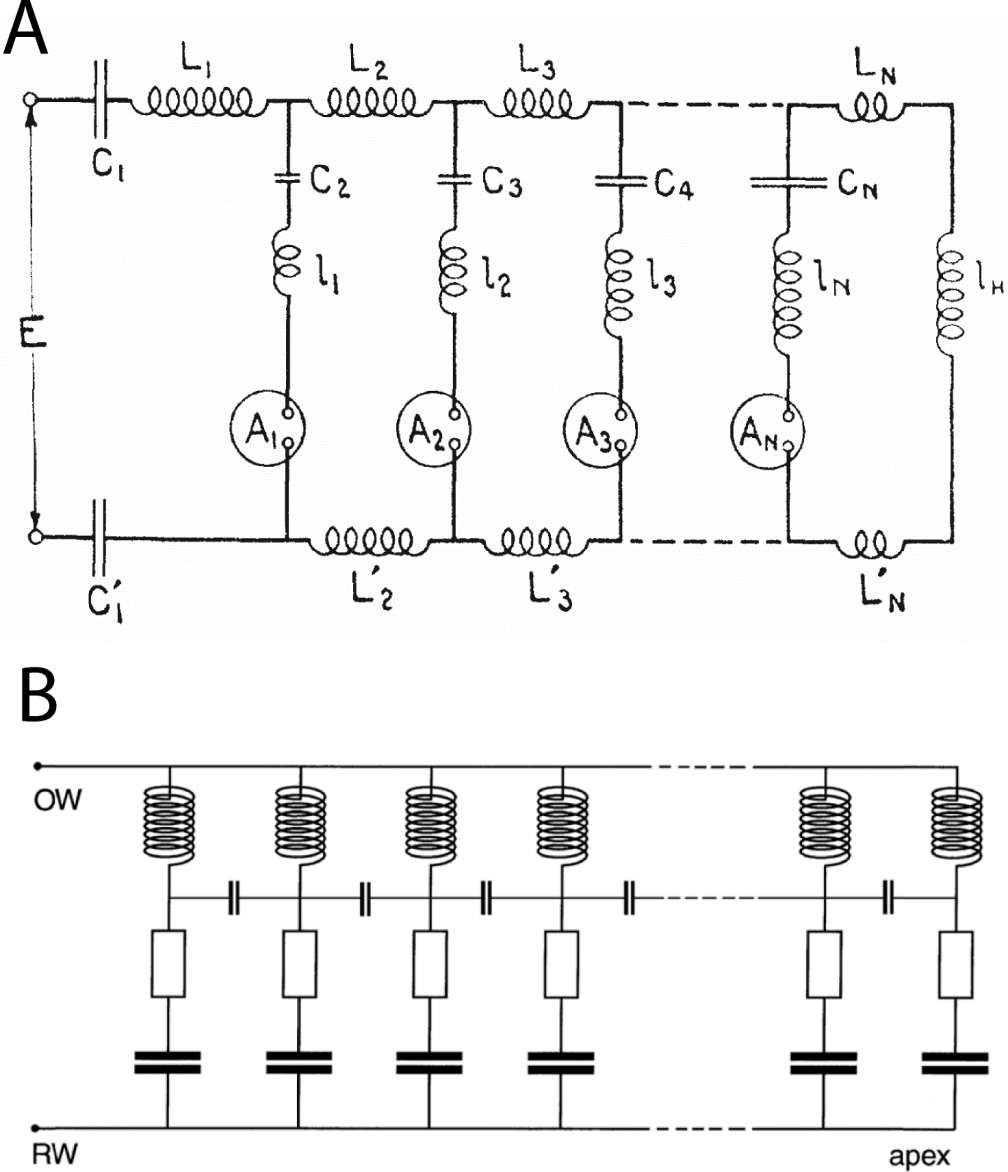
Equivalent circuits of the transmission line and the vibrating reed systems. (A) Transmission line model of the cochlea as first proposed by Wegel & Lane (1924). The stimulus travels through the resonant stages in series. The circles represent current detectors (nerve cells). (B) Equivalent circuit of the vibrating reed frequency meter, with the signal rail connected directly to all the resonant stages and a small capacitor reactively coupling each stage to its neighbours. The stimulus drives all stages in parallel, giving direct and stronger off-frequency forcing than the transmission line. The vibrating reed system is effectively a coupled filterbank model of the cochlea. Image credits: A, from Wegel & Lane (1924), The auditory masking of one pure tone by another and its probably relation to the dynamics of the inner ear, *Physical Review* 23:266–285; B, from Bell (2012), A resonance approach to cochlear mechanics, *PLOS One* 7:e47918.

These features are similar to those derived from standard cochlear models based on the transmission line (Duifhuis, 2012; Shera, 2015; Verhulst, Dau & Shera, 2012; Zweig, 2015), even though the two systems are structurally different. From comparing the equivalent circuit of the transmission line with that of the vibrating reed system (Fig. 16), it is clear that a major difference is that a stimulus frequency passes through the transmission line in series (Altoè, Pulkki & Verhulst, 2014; Duifhuis, 2012), whereas in the vibrating reed system the stimulus is applied to all stages in parallel. The key differences between serial and parallel systems are set out more fully in Bell (2012), where a comparison is made between the two types of pendulum model devised by Békésy, one in which the stimulus is delivered sequentially (the transmission line or traveling wave model) and another where the stimulus is delivered simultaneously (the resonance model). Both can produce a similar result, most notably spectral analysis and a traveling wave, although the underlying causal chain is not the same. Since the standard basis of cochlear mechanics is almost invariably the transmission line (Allen, 1980; Altoè, Pulkki & Verhulst, 2014; De Boer, 1996; Peterson & Bogert, 1950; Shera, 2015), it might be argued that the vibrating reed model has little relevance to the cochlea.

However, such a move might be an overreaction, and runs the risk of throwing the baby out with the bathwater. A recent paper (Zweig, 2015) makes the point that “the trick in modelling a complex system is to find a simpler system, and identify within it properties or relationships that hold in reality” (p. 1115). In this respect, the vibrating reed system is noteworthy because it can be represented with a single equation (Eq. (1)) yet gives rise to a wide range of features that conventionally emerge from more complex mathematics (more than 96 equations in the case of Zweig, 2015). Another notable aspect of Zweig (2015), one that encourages fresh interpretations, is that it begins with the inversion of actual cochlear data (the monkey data of Rhode, 1971), making it a phenomenological model which, in Zweig’s words, simply describes what the cochlea does, not how it does it (so that it “allows the data to speak first, not last”, p. 1115). Significantly, both Rhode and Zweig see a secondary peak beyond the main peak (which Zweig describes as a dip since he focuses on the region between the main and secondary peaks). Zweig calls the dip “dramatic” but “obscure” in origin. In this context, the appearance of a similar secondary peak in the vibrating reed system—in both cases appearing at a frequency ratio of about 1.1—invites using Eq. (1) as a simple but powerful phenomenological model. Zweig recognises that two models can have similar outputs for similar inputs, even though their underlying biological or physical structures differ. They can have feedback, feed-forward, or electrical forces, he observes, but the data will necessarily be blind to such internal operations, and even to the number of dimensions involved. The same pragmatism suggests that Eq. (1) could be used as a predictive tool, noting that the vibrating reed system can produce phenomena similar to those derived via more complex machinery (Shera, 2015). In his recent paper, Shera employs the coherent reflection model to generate ripples and frequency plateaus, features that emerge automatically from the vibrating reed system. Hence, the simplicity of the local oscillator model becomes a major virtue, not a limitation, and it avoids the need to introduce scattering and coherent reflection.

These broad perspectives encourage a closer look at the largely unexplored vibrating reed system. Several aspects are worth addressing.

### Filterbank models of the cochlea

Filterbank models of the cochlea have been a recurrent theme in cochlear mechanics (Lopez-Poveda, 2005), and it is of interest that many of them—those that assume all filters share a common input (*ibid*. p. 32)—are in broad alignment with the vibrating reed picture in which each filter corresponds to an individual reed. In such a context, further investigation of the resonance-like behaviour of the vibrating reed system could be illuminating (Kroeker, 2014).

However, in terms of the underlying physics, making an analogy between the cochlea and a filterbank requires careful assessment of how to interpret measured time delays, in particular “traveling wave delays”. Mechanically, the cochlea may, as noted by Zweig (2015), be viewed as a set of coupled harmonic oscillators driven by the instantaneous pressure difference across the partition. At the same time, Zweig concludes that a short-wavelength model with instantaneous interactions can behave like a long-wavelength model with nonlocal time delays.

The vibrating reed system is a local oscillator system driven instantaneously, so traveling wave delays need to be appropriately interpreted. It is possible to view the delays as group delays derived from tuned resonators (Bell, 2012), giving a similar outcome to that produced by sequential basilar membrane displacements (traveling waves). This still leaves open the question of what is the initial cochlear stimulus: is it bending of stereocilia due to instantaneous pressure differences, as conventionally thought, or is it perhaps outer hair cells acting as pressure sensors and responding to common-mode fluid pressure—an alternative but speculative mechanism (Bell, 2012; Bell, 2014). The advantage of the pressure sensor model is that it has both instantaneous parallel inputs (pressure-sensitive cells reacting to the fast pressure wave) and instantaneous parallel outputs—assuming that the activity of a cell translates, via a reciprocal volume change (Wilson, 1980), to an otoacoustic emission. There would then be a formal alignment between the vibrating reed system and the cochlea, an idea consistent with the Helmholtz picture of resonance (tentatively raised by Recio-Spinoso & Rhode, 2015) but the physics of the situation needs further investigation. The possibility of otoacoustic emissions being carried by fast pressure waves has been widely discussed, but not settled (He & Ren, 2013; Meenderink & Van der Heijden, 2010; Olson, 2013; Ren & Nuttall, 2006; Shera, 2006; Shera et al., 2007).

The above discussion returns us once again to the issue raised by Békésy about which path the stimulus energy takes before exciting a hair cell (Fig. 2). Does sound energy stimulate the hair cells simultaneously (in parallel) or does it create a serial traveling wave on the basilar membrane that has its own causal power (Ren, He & Porsov, 2011; Van der Heijden & Versteegh, 2015; Versteegh & Van der Heijden, 2013)? Ren and colleagues explicitly say that a wave at the apex *results from* vibrations at basal locations, while Versteegh & Van der Heijden (2013) express the view that vibrations at each point on the membrane are cumulatively *passed on* to the next point. Similarly, oscillator models of the cochlea also assume, in analogy to the transmission line, that the preferred energy route is along the chain (Van den Raadt & Duifhuis, 1990; Van Hengel, Duifhuis & Van den Raadt, 1996). A number of things could be said on this aspect, but there is one key point which emerges from Van der Heijden & Versteegh (2015). Based on observations of the gerbil cochlea, this recent paper estimates the energy flux at the peak and finds it is no more than 1 dB greater than the power at the middle ear, even for soft sounds. In other words, they conclude there is no cochlear amplifier. Such a startling finding invites the interpretation that power is being resonantly transferred through the fluid, not along the membrane, and an apt model for such a process might be the vibrating reed frequency meter. Figure 12 clearly demonstrates that most of the energy stimulating a particular reed, i.e., a hair cell, does so through external forcing—which means via sound pressure in the surrounding fluid.

At this point, however, there is a specific difficulty in trying to apply the vibrating reed analogy to the cochlea: the form of the coupling. In the vibrating reed system the coupling is elastic, whereas in the case of the cochlea coupling is usually considered to occur via fluid mass, and this aspect is now discussed.

### Forms of coupling and implications

The vibrating reed system has reactive (capacitive) coupling between each of the tuned elements, contributed by the elasticity of the rubber band (*κ* in Eq. (1)). Note that *κ* is a real quantity, although further investigations might involve making *κ* complex, a scheme used by Vilfan & Duke (2008) to introduce different forms of coupling. In the cochlea, elastic coupling is normally assumed negligible (Appendix B of Mammano & Nobili, 1993; Meaud & Grosh, 2010); however, some investigations have involved looking at the effects of various forms of coupling, and the issue still needs to be resolved (Jaffer, Kunov & Wong, 2002; Kim et al., 1980; Meaud & Grosh, 2010; Nam, 2014; Ni et al., 2014; Wickesberg & Geisler, 1986).

In the vibrating reed system, coupling is strictly nearest-neighbour so that longer range (or global) coupling is absent. Standard cochlear models normally specify long-range coupling via the fluid (but see Nam, 2014), so comparisons here may be illuminating. In particular, they could help decide the question, raised above, of whether energy emerges from the cochlea via fast compression waves or slow traveling waves.

One recent investigation of oscillator coupling in a cochlear model (Fruth, Jülicher & Lindner, 2014) concludes that nearest neighbour coupling plays a primary role in governing the global dynamics of the cochlea. Fruth and colleagues found that when suitable coupling constants (elastic and hydrodynamic) were used in an active model, spontaneous otoacoustic emissions (SOAEs) occurred at a preferred frequency ratio of 1.06. As the coupling was made stronger, the oscillator clustering and the favoured inter-emission SOAE spacing steadily increased, in line with the passive Frahm reed modelling done here. At this point, the form and range of coupling is left as subjects for further research.

### Effects in banks of discrete oscillators

The present modelling has used a set of passive, coupled harmonic oscillators. Only one other investigation (Authier & Manley, 1995) has used discrete passive oscillators to model resonating elements in the inner ear, and in that study of the lizard ear the authors decided not to investigate coupling in detail because of its mathematical complexity. At the same time, there have been numerous works that have studied the general case of externally forced coupled oscillators (see Pikovsky, Rosenblum & Kurths, 2001 and references in Bell, 2012). A common finding is clustering and the formation of frequency plateaus. In any coupled system subject to external forcing, each oscillator is faced with two conflicting tendencies: to synchronise with the external force (forced entrainment) or with its neighbours (mutual entrainment); as explained by Pikovsky, the compromise is to form frequency plateaus (*op. cit.*, p. 126).

As applied to the cochlea, however, the discrete oscillator approach is not common. Pioneering work on modelling the cochlea with discrete active elements and solving it in the time domain was originally done by Duifhuis and colleagues (Duifhuis et al., 1986). More recently, a range of discrete formulations using active elements have appeared (Altoè, Pulkki & Verhulst, 2014; Epp, Verhey & Mauermann, 2010; Fruth, Jülicher & Lindner, 2014; Verhulst, Dau & Shera, 2012). In these works, oscillators were made active by adjusting the effective damping parameter to be negative rather than positive, the motive being to understand spontaneous otoacoustic emissions (SOAEs).

A difficulty with all active models is the problem of separating the contribution of an active oscillator’s internal dynamics (typically that of a van der Pol oscillator) from that of system-wide properties (Duke & Jülicher, 2003; Gelfand et al., 2010; Vilfan & Duke, 2008; Wit & Van Dijk, 2012; Wit, Van Dijk & Manley, 2012). The virtue of a completely passive system like the vibrating reed model is that it avoids that particular complication.

### Secondary peaks and ripples

An inherent feature of the coupled vibrating reed system is the formation of a secondary peak beyond the main peak (Figs. 5–7). This feature was first observed by Wilson, and the simulations done here confirm its presence. In the spatial dimension, the secondary peak occurs at a point whose natural frequency is about 1.1 times lower than that of the main peak, whereas in terms of driving frequency, the secondary peak occurs at a frequency about 1.1 times higher. The exact ratio depends on the coupling strength, as shown in Fig. 8. With the finer resolution provided by 201 reeds, the simulations confirm that the secondary peak appears some 55–90 Hz beyond the 1.7–1.8 kHz primary peak (a ratio of about 1.05 for *κ* = 200), and, intriguingly, also show it accompanied by a set of smaller, closely spaced ripples (Figs. 8–11).

Both the secondary peak and the ripples are of interest because sometimes fine-grained and periodic structure is observed in the cochlea, seen in the audiogram (Elliott, 1958), basilar membrane measurements (Olson, 2001; Rhode, 1971), and otoacoustic emissions (Braun, 1997; Braun, 2013; Fruth, Jülicher & Lindner, 2014; Kemp & Chum, 1980; Lineton & Lutman, 2003).

A secondary peak can sometimes be seen in transmission line models, although at small amplitude (Allen, 1977; Allen & Sondhi, 1979; Neely, 1981; Zweig, 2015). As noted earlier, the model of Zweig (2015) was constructed by inverting the cochlear data of Rhode (1971), so he regards the secondary peak as an important feature that requires explanation. Ripples on the low-frequency side of spatially based frequency responses have been observed in a number of discrete active oscillator models (Altoè, Pulkki & Verhulst, 2014; Epp, Verhey & Mauermann, 2010; Verhulst, Dau & Shera, 2012), producing response curves with irregular features not unlike those seen here.

Although the ratio between the main and secondary peak depends on the coupling constant, values of around 1.05 were commonly seen using the arbitrary parameters adopted in this work. Coincidentally, this ratio is in keeping with the approximate 1.05 steps seen in Fig. 6 of Shera (2015) and with small spatial irregularities found in related work (e.g., Temchin & Ruggero, 2014 in the chinchilla). The ratio between successive ripples in the present work turned out to be somewhat smaller (Figs. 9–11 and 13–15), and typically 1.03 for *κ* = 200, giving a periodicity of 30–50 Hz over the mid-frequency range examined (Figs. 9 and 13). The periodic variations might be related to the behaviour of stimulus frequency otoacoustic emissions, in which cyclic phase variations are observed when a stimulating tone is swept in frequency (Kemp & Chum, 1980; Lineton & Lutman, 2003; Shera, 2003).

Shera (2015) explains the ripples (his Fig. 2B) in terms of coherent reflection between an active region and the base. Recirculating wave energy is also assumed in the models of Van Hengel, Duifhuis & Van den Raadt (1996), Epp, Verhey & Mauermann (2010), and Verhulst, Dau & Shera (2012), who underline the importance of matching the middle ear impedance to that of the cochlear base. Shera finds that his active transmission line model generates not only ripples but also sets of frequency plateaus that he describes as a cochlear staircase. Whereas the staircase apparently depends on a combination of micromechanical irregularities and wave backscattering, it is significant that similar frequency plateaus can be generated by a simpler resonance-based arrangement in which the frequency plateaus are locally produced (Fig. 15C).

## Conclusion

The vibrating reed frequency meter is a simple system which can generate an extensive range of interesting features. Numerical modelling has confirmed that the system reproduces some well-known aspects of cochlear mechanics, and the detail available from computer simulation well exceeds that which can be realised with physical models. Modelling has revealed fine-grained properties such as a secondary peak and an associated set of ripples, features which have only recently been clearly identified and ascribed to more complex cochlear processes (Shera, 2015; Zweig, 2015).

Zweig’s paper notes that two models can “match” (have similar outputs for similar inputs), despite having different biological or physical structures (p. 1116 of Zweig, 2015). In this sense, the differences between the vibrating reed system and the traditional transmission line model may not be as important as first appears. What effect does it make that the reeds in the vibrating reed system are driven simultaneously (in parallel) by the oscillating magnetic field surrounding them, rather than sequentially? What is the effect of having strictly local coupling which is due to elasticity, not mass? Further investigation of these questions could be rewarding. A major advantage of the vibrating reed system is that it is a simple passive system whose behaviour is easy to understand. Perhaps nearest-neighbour elastic coupling may have a direct role in local interactions between portions of the basilar membrane. No doubt refinements are needed, but provided the limitations are kept in mind, aspects of the vibrating reed system—like those which first attracted Békésy and Wilson—may spark renewed interest in this intriguing system.

## Additional Information and Declarations

### Data Availability

The following information was supplied regarding data availability: All results presented in this manuscript can be reproduced by performing numerical calculations according to Eq. (1).
